# COVID-19 through Adverse Outcome Pathways: Building Networks to Better Understand the Disease – 3^rd^ CIAO AOP Design Workshop

**DOI:** 10.14573/altex.2112161

**Published:** 2022-01-03

**Authors:** Laure-Alix Clerbaux, Núria Amigó, Maria João Amorim, Anna Bal-Price, Sofia Batista Leite, Anna Beronius, Gillina F. G. Bezemer, Ann-Charlotte Bostroem, Annamaria Carusi, Sandra Coecke, Rachel Concha, Evangelos P. Daskalopoulos, Francesca Debernardi, Eizleayne Edrosa, Steve W. Edwards, Julija Filipovska, Natàlia Garcia-Reyero, Felicity N. E. Gavins, Sabina Halappanavar, Alan J. Hargreaves, Helena T. Hogberg, Mylène T. Huynh, Daniel Jacobson, Jonathan Josephs-Spaulding, Young Jun Kim, Hyun Joon Kong, Catharine E. Krebs, Ann Lam, Brigitte Landesmann, Adrienne Racanelli Layton, Yong Oh Lee, Donna S. Macmillan, Alberto Mantovani, Luigi Margiotta-Casaluci, Marvin Martens, Rosalinde Masereeuw, Sally A. Mayasich, Liang Merlin Mei, Holly Mortensen, Amalia Munoz, Penny Nymark, Elan Ohayon, Joshi Manoj Ojasi, Alicia Paini, Nikolaos Parissis, Surat Parvatam, Francesca Pistollato, Magdalini Sachana, Jorid Birkelund Sørli, Kristie M. Sullivan, Jukka Sund, Shihori Tanabe, Katya Tsaioun, Mathieu Vinken, Laura Viviani, Jennifer Waspe, Catherine Willett, Clemens Wittwehr

**Affiliations:** 1 European Commission, Joint Research Centre, Ispra, Italy; 2 Biosfer Teslab, Reus, Spain; 3 Instituto Gulbenkian de Ciência, Lisbon, Portugal; 4 Institute of Environmental Medicine, Karolinska Institutet, Stockholm, Sweden; 5 Impact Station, Hilversum, The Netherlands; 6 Interchange Research, London, UK; 7 Fairleigh Dickinson University, Green Neuroscience Laboratory, San Diego, CA, USA; 8 Division of Otorhinolaryngology, Department of Biotechnologies and Life Sciences, University of Insubria, Ospedale di Circolo e Fondazione Macchi, Varese, Italy; 9 Green Neuroscience Laboratory, Neurolinx Research Institute, San Diego, CA, USA; 10 RTI International, Research Triangle Park, NC, USA; 11 Independent scientist, Ohrid, North Macedonia; 12 US Army Engineer Research and Development Center, Vicksburg, MS, USA; 13 The Centre for Inflammation Research and Translational Medicine (CIRTM), Brunel University London, London, UK; 14 Environmental Health Science and Research Bureau, Health Canada, Ottawa, Ontario, Canada; 15 School of Science and Technology, Nottingham Trent University, Nottingham, UK; 16 Johns Hopkins Bloomberg School of Public Health, Baltimore, MD, USA; 17 Department of Preventive Medicine and Biometrics, Uniformed Services University of the Health Sciences, Bethesda, MD, USA; 18 Biosciences, Oak Ridge National Laboratory, Oak Ridge, TN, USA; 19 Institute for Experimental Medicine, Kiel University, Kiel, Germany; 20 Korea Institute of Science and Technology Europe Forschungsgesellschaft mbH, Saarbrücken, Germany; 21 University of Illinois at Urbana-Champaign, Champaign, IL, USA; 22 Physicians Committee for Responsible Medicine, Washington, DC, USA; 23 US Consumer Product Safety Commission, Bethesda, MD, USA; 24 Humane Society International, Washington, DC, USA; 25 Istituto Superiore di Sanità, Rome, Italy; 26 Department of Bioinformatics - BiGCaT, NUTRIM, Maastricht University, Maastricht, The Netherlands; 27 Utrecht Institute for Pharmaceutical Sciences, Utrecht University, Utrecht, The Netherlands; 28 University of Wisconsin-Madison Aquatic Sciences Center at US EPA, Duluth, MN, USA; 29 US EPA, Durham, NC, USA; 30 European Commission, Joint Research Centre, Geel, Belgium; 31 Hiranandani College of Pharmacy, Mumbai, India; 32 Centre for Predictive Human Model Systems Atal Incubation Centre - Centre for Cellular and Molecular Biology Habsiguda, Hyderabad, India; 33 Environment Health and Safety Division, Environment Directorate, Organisation for Economic Cooperation and Development (OECD), Paris, France; 34 The National Research Centre for the Working Environment, Copenhagen, Denmark; 35 Division of Risk Assessment, Center for Biological Safety and Research, National Institute of Health Sciences, Kawasaki, Japan; 36 Department of Pharmaceutical and Pharmacological Sciences, Vrije Universiteit Brussel, Brussels, Belgium; 37 Sheffield Teaching Hospitals, Sheffield, UK

## Abstract

On April 28–29, 2021, 50 scientists from different fields of expertise met for the 3^rd^ online CIAO workshop. The CIAO project “Modelling the Pathogenesis of COVID-19 using the Adverse Outcome Pathway (AOP) framework” aims at building a holistic assembly of the available scientific knowledge on COVID-19 using the AOP framework. An individual AOP depicts the disease progression from the initial contact with the SARS-CoV-2 virus through biological key events (KE) toward an adverse outcome, such as respiratory distress, anosmia or multiorgan failure. Assembling the individual AOPs into a network highlights shared KEs as central biological nodes involved in multiple outcomes observed in COVID-19 patients. During the workshop, the KEs and AOPs established so far by the CIAO members were presented and positioned on a timeline of the disease course. Modulating factors influencing the progression and severity of the disease were also addressed, as well as factors beyond purely biological phenomena. CIAO relies on an interdisciplinary crowdsourcing effort, therefore, approaches to expand the CIAO network by widening the crowd and reaching stakeholders were also discussed. To conclude the workshop, it was decided that the AOPs/KEs will be further consolidated, integrating viral variants and long COVID when relevant, while an outreach campaign will be launched to broaden the CIAO scientific crowd.

## 1 Introduction

### 1.1 The CIAO project

Coronavirus disease 2019 (COVID-19) is an ongoing global health emergency. Researchers around the world have mobilized to investigate the biological mechanisms of the disease, resulting in a plethora of data being generated daily. The CIAO project aims to make sense of all the scientific knowledge on COVID-19 by exploiting the adverse outcome pathway (AOP) framework (Nymark et al., 2021). The AOPs may not necessarily produce original data, but, based on published work, depict the causal relationships that link an initial perturbation over a series of biological key events (KE) toward an adverse outcome (AO), such as respiratory distress or multiorgan failure. AOPs are living documents, in the sense that they can be continuously updated as new information becomes available. AOPs covering a wide variety of AOs have already been developed and are stored in the open access AOP Wiki^1^. Thus, AOPs provide a platform for organizing, revising, and consolidating the abundant and fast-evolving scientific knowledge on COVID-19 at different biological levels. In addition, they leverage knowledge gained from other fields of research, such as toxicology, to describe the viral disease based on a mechanistic understanding (Kim et al., 2021; Kinaret et al., 2020; Nymark et al., 2021; Vinken, 2013). Moreover, such organization of knowledge helps to capture the various factors influencing clinical outcomes. Finally, the modular aspect of AOPs allows the development of AOP networks where shared KEs become evident (Villeneuve et al., 2019, 2014). This is particularly interesting for COVID-19, as the clinical outcomes are disparate, while interconnected KEs may identify central biological mechanisms involved in multiple AOs.

Building an AOP network modelling COVID-19 pathogenesis relies on interdisciplinary collaborative effort, synergizing exchange between experts from different fields to translate complex biology into messages understandable across disciplines. The CIAO project aims at harnessing the power of crowdsourcing via the AOP Wiki platform to provide understandable knowledge about the biological mode of action of the virus that could then support policy and healthcare decisions. Currently, more than 65 scientists from 40 organizations around the world are participating in the project, which is steered by the European Commission, Physicians Committee for Responsible Medicine (PCRM) and Humane Society International (HSI).

On October 1–2, 2020, and January 27–28, 2021, the first two online CIAO workshops were held (^2^, Wittwehr et al., 2021). Seven working groups (WG) emerged from the second workshop, (i) the Hub AOP WG, focused on investigating KEs common to multiple COVID-19 AOs, (ii) the Lung AOP WG, dealing with pulmonary-related AOPs, (iii) the Other Organs AOP WG, focusing on building AOPs relevant to several organs, (iv) the Neuro AOP WG on investigating COVID-19 AOPs associated with neurological syndromes, (v) the Modulating Factors (MF) WG, examining the biological factors that influence the COVID-19 outcomes, (vi) the Multiscale Impact WG, focusing on the development of AOPs beyond how SARS-CoV-2 affects the organism of infected individuals, and (vii) the Literature Review WG on covering various approaches of systematic literature review to support AOP development. On April 28–29, 2021, the 3^rd^ CIAO workshop was held with 50 participants over 2 half-days. The workshop was facilitated by Laure-Alix Clerbaux, Laura Viviani and Clemens Wittwehr.

### 1.2 Goals of the 3^rd^ CIAO AOP Design Workshop

**Fig. 1:**
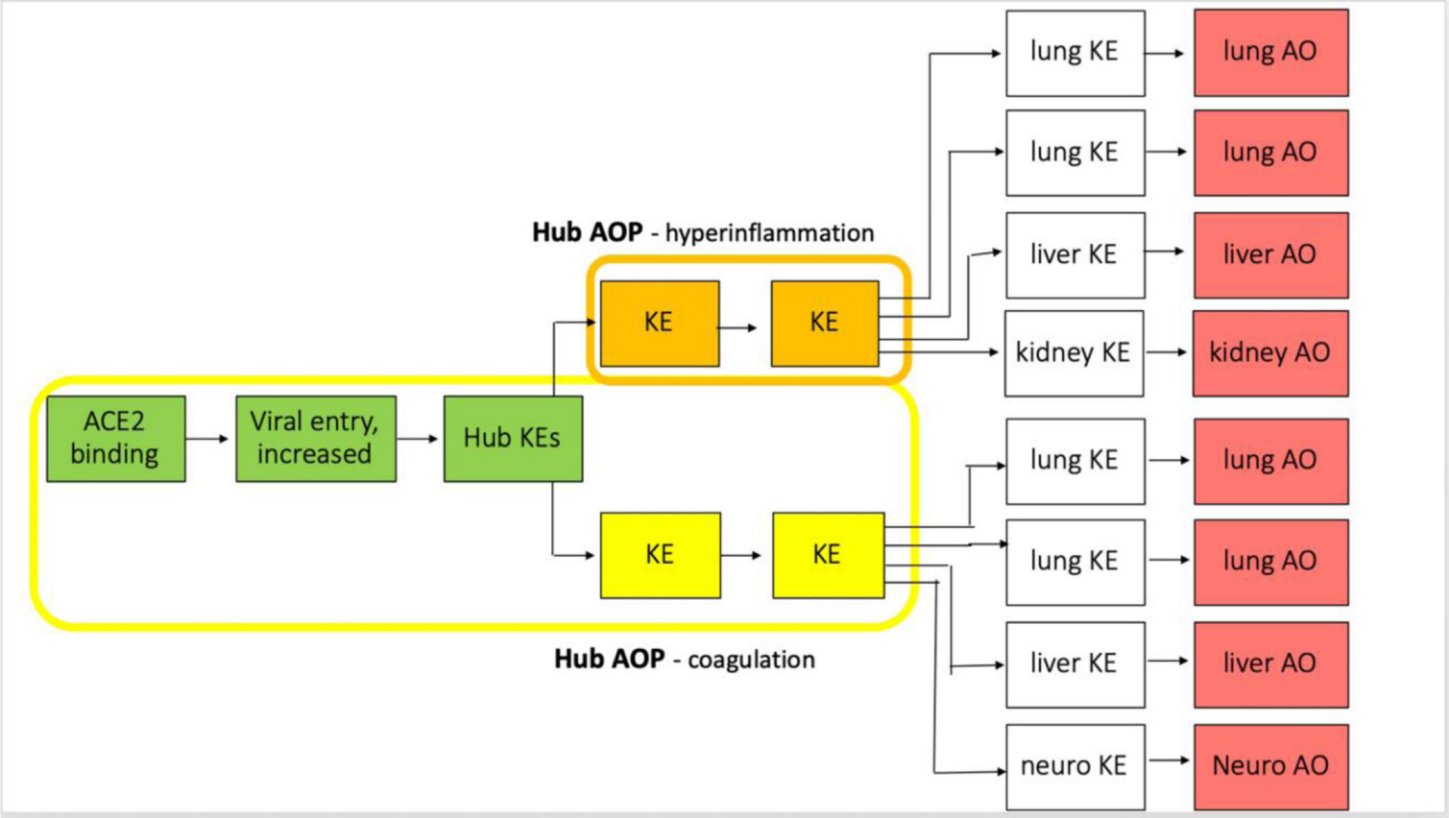
Schematic representation of the *COVID-19 AOP network* built on Hub KEs (ACE2 binding, viral entry, coronavirus production and ACE2 dysregulation) and Hub AOPs (hyperinflammation and coagulation), and leading to AOs in various organs

**Fig. 2:**
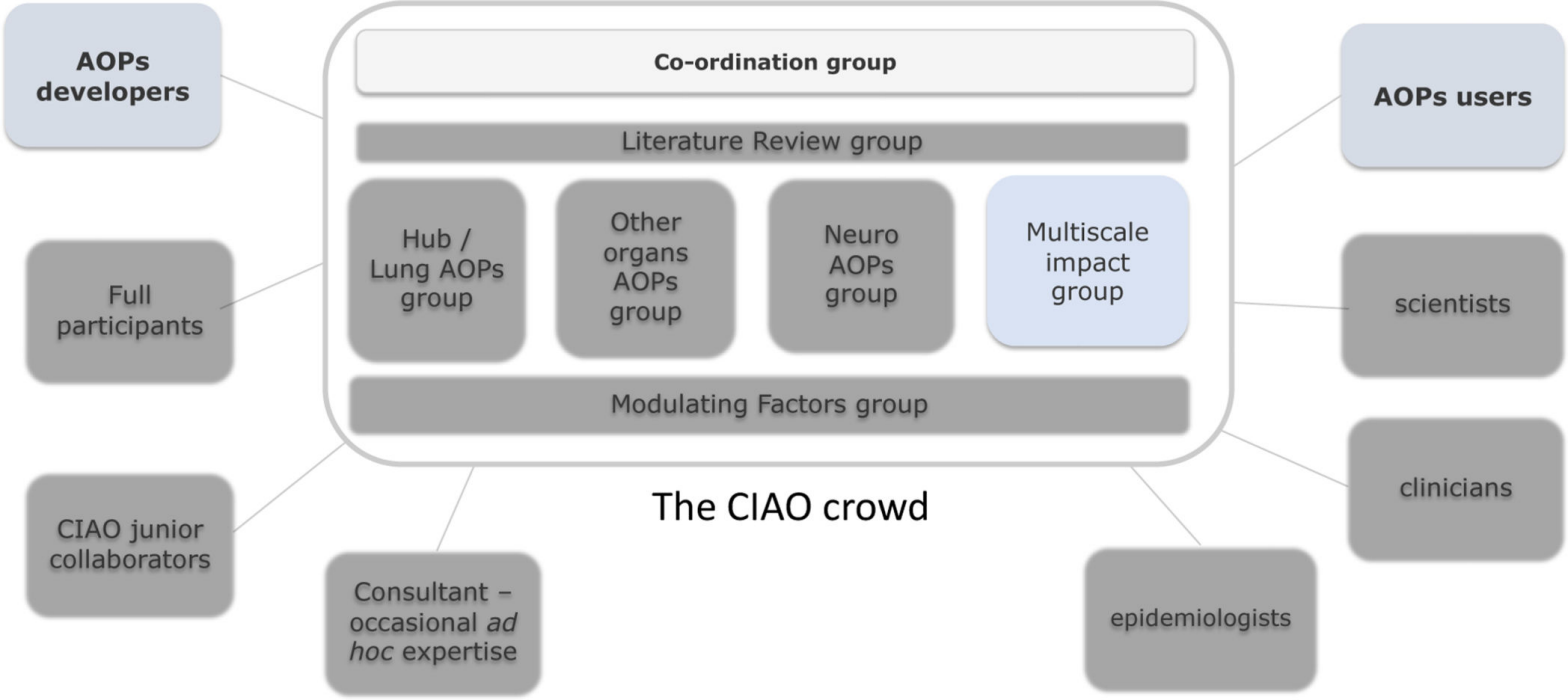
Schematic representation of the *CIAO network* AOPs developers encompass full participants at one of more working group, CIAO junior collaborators (master or PhD student), consultants offering occasional *ad hoc* expertise. AOPs users could be scientists, clinicians, epidemiologists among others.

After welcoming words from Maurice Whelan, Head of the Chemical Safety and Alternative Methods Unit at the Joint Research Centre of the European Commission, Laure-Alix Clerbaux presented the goals to be achieved during this workshop. The first goal was to gather and share the scientific achievements in terms of AOPs/KEs developed so far by the various WGs and set the scene for developing a “COVID-19 AOP network”. The early KEs such as binding of the virus to the ACE2 receptor and viral entry (green) are obviously common to all COVID-19 AOs. Furthermore, two series of KEs including those initial events and leading to coagulation (yellow) or hyperinflammation (orange) respectively, were identified as central and preceding multiple organ-specific KE (white) and COVID-19 AOs (red). Therefore, building an AOP network depicting COVID-19 would be done by using shared KEs as exchangeable building blocks (Fig. 1). The second goal of the workshop was to define strategies to expand the CIAO network by broadening the crowd via new ways of collaboration (AOP developers), as well as by reaching out more efficiently to the CIAO target audience (AOP users) (Fig. 2).

### 1.3 Publication strategy

**Fig. 3:**
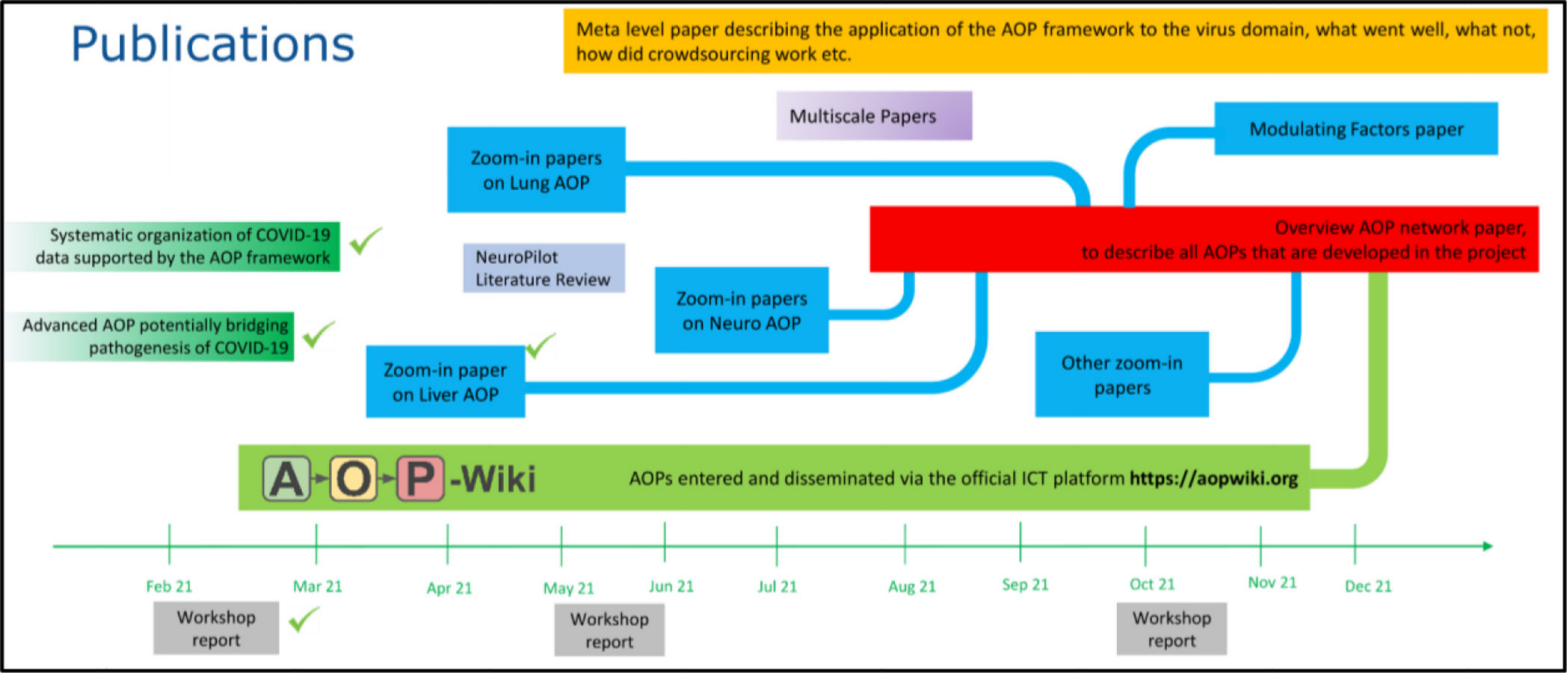
The CIAO publication strategy

A presentation by Sofia Batista Leite on the communication and data-sharing platforms used in the project (Google drive, Slack^3^, Zotero^4^) and the release of a recent bimonthly internal CIAO newsletter followed. Subsequently, the CIAO publication strategy was discussed. A subgroup to plan the CIAO publications (content, sequence, authors) had been formed before the workshop, and Clemens Wittwehr presented the results of the first meeting of this group. It had been agreed that the two main publications will be (1) a high-level overview of the AOP network integrating all AOPs developed in the project and entered into the Wiki (red in Fig. 3) and (2) a meta-level paper describing how the AOP framework and the crowdsourcing effort were applied to the COVID-19 domain (in yellow). Papers on individual aspects of the CIAO project, such as various in-depth AOP descriptions and modulating factors (in blue), a neuro-related pilot literature study, and the multiscale approach (in purple) are also foreseen.

## 2 Scientific outcomes: building an AOP network depicting COVID-19

Each group presented the major achievements in terms of AOPs and KEs developed and entered into the AOP Wiki over the last three months (Annex A^5^).

### 2.1 Hub-Lung WG

The Hub-AOP and Lung-AOP WGs, led by Penny Nymark and Maria João Amorim, joined forces after the second workshop to work synergistically. The overall aim of the joint WG was to focus on the development of AOPs describing lung injuries and functioning as a case study for the development of overarching Hub KEs. Subsequent development of Hub KEs can then include extensions for application to other organs. The work has resulted in the development and/or refinement of 10 new or previously developed AOPs in the AOP Wiki (new AOPs: 377, 378, 379, 382, 385, 392; previously developed AOPs: 173, 302, 319, 320), as well as a new stand-alone KE (KE 1857) and a KE in development (interferon-I antiviral response antagonism) (Tab. 1). The KEs/AOPs cover a range of mechanisms, including three Hub KEs representing viral entry and viral production in infected cells, ACE2 dysregulation (KE1854), Hub AOPs covering oxidative stress, coagulation, thrombosis, bradykinin and fibrinolytic dysregulation, hyperinflammation, toll-like receptor dysregulation as well as pulmonary-related AOs including acute respiratory distress syndrome (ARDS), ARDS-related mortality, lung fibrosis and impaired lung function.

All AOPs and KEs therein are available for further refinement to become suitable for description of other AOs. The KEs developed by the Hub-Lung group are central for many SARS-CoV-2 related AOPs and, therefore, there is a pressing need to finalize their inclusion in the AOP Wiki.

### 2.2 Other Organs WG

The Other Organs WG, coordinated by Kristie Sullivan, emphasized the differences between direct and indirect AOPs. The Hub KEs and Hub AOPs initiate the indirect AOPs. The AOPs from this WG (Tab. 2) still need to be defined and entered into the AOP Wiki (TBD). Mathieu Vinken presented three AOPs depicting the pathology-related mechanisms underlying the hepatic impact of COVID-19. Two AOPs depict the indirect pathways induced by the binding of SARS-CoV-2 to lung ACE2 receptors and involving the Hub AOPs on hyper-inflammation and thrombosis, ultimately affecting the liver. The third AOP describes the direct pathway triggered by the binding of the virus to cholangiocyte ACE2 receptors. Evangelos Daskalopoulos then presented the cardiovascular AOPs. This proposed AOP describes the involvement of the RAAS in the development of noxious effects in the heart, mediated by ACE2 downregulation. More specifically, ACE2 downregulation following SARS-CoV-2 infection drives the attenuation of the Angiotensin(1–7)/MAS receptor pathway and the enhancement of the Ang-II/AT1 receptor pathway, leading to the development of deleterious pro-inflammatory, pro-thrombotic and pro-hypertrophic effects in the myocardium. No new inputs were presented from the kidney at this WS. Finally, Laure-Alix Clerbaux proposed putative intestinal AOPs. ACE2 receptors are highly expressed in enterocytes and play key roles in renin-angiotensin balance as well as in the amino acid intestinal homeostasis. ACE2 dysregulation is proposed to lead to intestinal hyperpermeability, resulting in gastrointestinal (GI) disorders as evidenced by diarrhea, nausea and vomiting observed in many COVID-19 patients. Besides, similarly to the liver, heart and kidney, systemic coagulation and hyperinflammation (Hub AOPs) leads to GI complications in COVID-19.

### 2.3 The Neuro-AOP WG

The Neuro-AOP WG, coordinated by Magdalini Sachana and Helena Hogberg, worked toward: i) refining the titles of KEs, ii) developing key event relationships (KERs) following the OECD Users’ Handbook^6^ for developing and assessing AOPs and iii) exchanging experience in AOP development. Some of the initial KEs identified at the January workshop (e.g., sustentacular cell regeneration, regeneration of olfactory neurons and neuroepithelial cells) will not be described as separate KEs. These KEs will instead be considered feedback loops because they do not play a direct causal role in the AOP but act as key compensatory mechanisms that contribute to how severely the KE upstream needs to be impacted in order to affect the KE downstream. For this reason, the information about these specific KEs will be described as part of the quantitative understanding section of the KER description. The work has resulted in the development of four new AOPs, and three of them are already available in the AOP Wiki (Tab. 3). These AOPs lead to the major AOs that have been associated with the effects of SARS-CoV-2 on the nervous system (i.e., anosmia, encephalitis, stroke and epilepsy). Reports on multiple sclerosis and long-term neuronal effects are also of interest to the Neuro-AOP WG, and evidence is being explored further. The WG also reported on the challenges encountered to incorporate MFs, as they might be important for the KE itself and not only for documentation of the KER. Furthermore, the lack of clear mechanistic *in vitro* or *in vivo* data complicates the AOP process. However, as new studies get published, this will likely be enhanced. Looking forward, the WG plans to start publishing the collected knowledge, identify scientific gaps in research, increase the impact by linking collected knowledge to therapeutic interventions, and mapping various factors that can modulate the AOPs related to the nervous system and that are triggered by SARS-CoV-2 infection.

### 2.4 Positioning the COVID-19 AOPs and KEs on the disease timeline

**Fig. 4:**
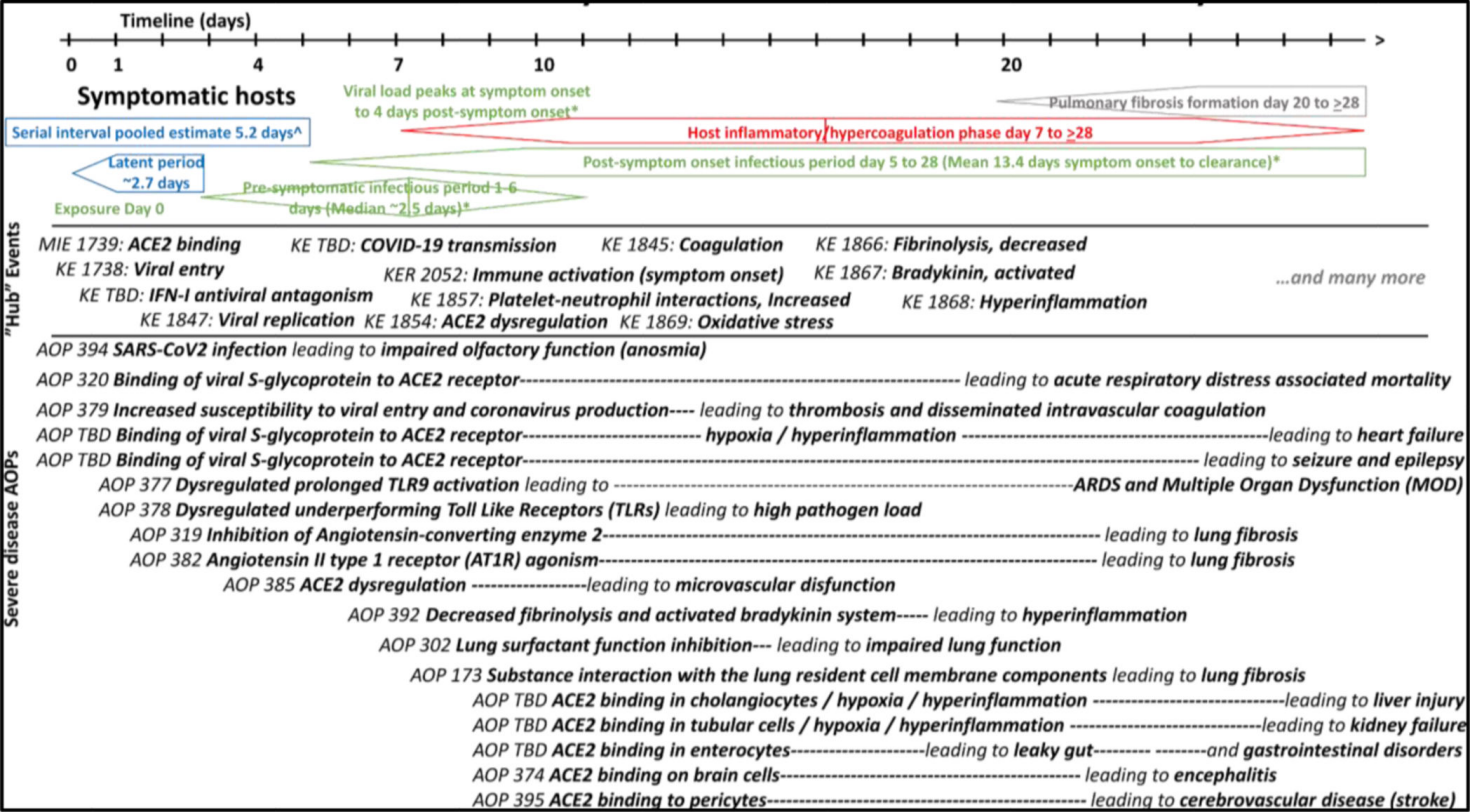
COVID-19 timeline KE and AOPs (Courtesy of Sally Mayasisch)

The AOPs and KEs developed were then positioned on the timeline of the course of the disease (Fig. 4). The COVID-19 disease timeline is a visualization based on current literature on the timing of disease phases, from exposure through pre-symptomatic infectious period, normal symptoms, dysregulated immune responses, and severe outcomes, to which the developed and developing KEs and AOPs have been aligned. Understanding the timing may help in organizing information within KEs, and KEs within AOPs. The viral entry KE and early KEs coincide with the time from exposure to symptoms, within which are the latent period (time from exposure to infectiousness) and the serial interval (time interval between the onset of symptoms in the primary and secondary case). Latent period calculation on the timeline is based on the serial interval and the median pre-symptomatic infectious period. Serial interval 5.2 days (Rai et al., 2021) minus 2.5 days pre-symptom infectious period (Byrne et al., 2020) ≈ 2.7 days. The latent period was longer in asymptomatic cases (4–9 days); presymptomatic transmission occurs from about 3 days after exposure to symptom onset at about day 5–7, viral load peaks from about day 5–7 to day 9–11, and the host can remain infectious until symptom clearance or death. Onset of symptoms at about 5–7 days coincides with the immune dysregulation beginning at about 7 days, and KEs including immune activation and ACE2 dysregulation. Subsequent hospital admission upon respiratory distress at about 7–10 days (Wang et al., 2020) coincides with the KEs hypoxia, hypercoagulation, and thrombosis. Those events then lead to severe AO (e.g., ARDS, multi-organ dysfunction, and lung fibrosis) starting around the 3^rd^ or 4^th^ week (Datta et al., 2020). The hyperinflammatory/hypercoagulation and pulmonary fibrosis formation phases on the timeline were put forth by Polak et al. (2020) from histopathology studies of 65 individual patients, corroborating other noted timelines.

### 2.5 Modulating Factors WG

The WG on Modulating Factors (MFs) first briefly presented the different factors the group had chosen to investigate based on their expertise, namely sex, age, vitamin D, diet, microbiota, lipid-related aspects, genetics, cardiovascular disease, drugs, air pollution, and chemicals such as per- and polyfluoroalkyl substances. Then the group more specifically presented how the MFs age (Mylène Huynh) and drugs (Nikolaos Parissis) might interfere with the clinical outcomes of COVID-19. Then, Brigitte Landesmann highlighted some challenges concerning the integration of modulating factors into the AOP framework and the AOP Wiki. According to current thinking and OECD Users’ Handbook guidance^6^, MFs alter the shape of the responseresponse function that describes the quantitative relationship between two KEs (i.e., within the KER), and they should be listed in the KER subsection “response-response relationship” along with relevant mechanistic information and solid evidence. However, the collected information indicates that in some cases MFs have an impact also on the KEs themselves. There is also an important time dimension with different impact, whether the modulating effect occurs prior to or concurrent with the infection, and that needs to be captured but is absent at present. There was a key discussion on how the AOP Wiki platform could be better suited to accommodate MFs. In the Wiki, information on MFs can be entered on KER pages and the AOP main page but not in the KE itself. Only life stage and sex applicability can also be indicated for KEs. Still, even for these parameters, there is no dedicated space for the description of necessary details. As such, significant differences between men and women cannot be specified, because entry is via a drop-down menu. In addition, different life-stages might have different impacts but are not strictly separable. One or more KERs might be differently affected by one or more MFs, and capturing this diversity in the overall AOP description in the Wiki is currently not facilitated or structured sufficiently. An additional paragraph describing the impact of MFs might be considered as free text as part of the overall assessment for the AOP. In summary, the WG output supports that MFs should be duly considered and described in the AOP Wiki.

### 2.6 Literature Review WG

The Literature Review WG, coordinated by Donna Macmillan, presented an introduction to literature reviews, highlighting key differences between narrative review, systematic review, and systematic scoping or mapping reviews – and when each is appropriate. As the body of literature surrounding COVID-19 and its related AOs is large and increasing by the day, a pilot project focusing only on neurological outcomes related to COVID-19 was initiated. The project began by downloading all COVID-19 literature available in PubMed (86,075 papers as of January 2021). After filtering those containing neurological keywords, about 10,000 papers remained. These papers were manually screened using Sciome’s Swift-ActiveScreener, and if a paper’s title or abstract referred to the neurological impact of COVID-19, this paper was reserved, leaving about 2,000 papers. The next step for the WG is to fully assess each of these papers. Any paper matching the predetermined exclusion criteria (e.g., no primary data, no neurological outcomes reported) will be filtered out, and the final set of papers will be used to publish a systematic scoping review on the neurological effects of COVID-19. The protocol will be published in due course and may provide a useful starting point for other WGs to undertake similar systematic scoping reviews.

### 2.7 Multiscale Impact Rogues WG

The Multiscale Impact Rogues WG, led by Ann Lam and Elan Ohayon, reported the outcomes of its five meetings and of various satellite discussions in chronological order of the meetings. The foundational ideas were outlined at the January CIAO workshop. The term “rogues” was proposed to reflect an act of rebellion against a molecular-centric perspective in the AOP field and the narrow outlook in the pandemic response. This is reflected in the current definitional assignments of MIE, KE, KER, and temporal assignment of factors. Although some of these themes are also explored in the other WGs, the anchor point remained a molecular mechanistic description without spatial and “higher-level” factor centrality. The group mandate aims to (a) elucidate the multiscale factors of COVID-19 and future pandemics prevention, (b) uphold the central goal of having a direct impact on resilience and outcomes for individuals and society, and (c) evolve the AOP framework to achieve understanding and impact across levels and time.

#### Collaborative investigations.

**Fig. 5:**
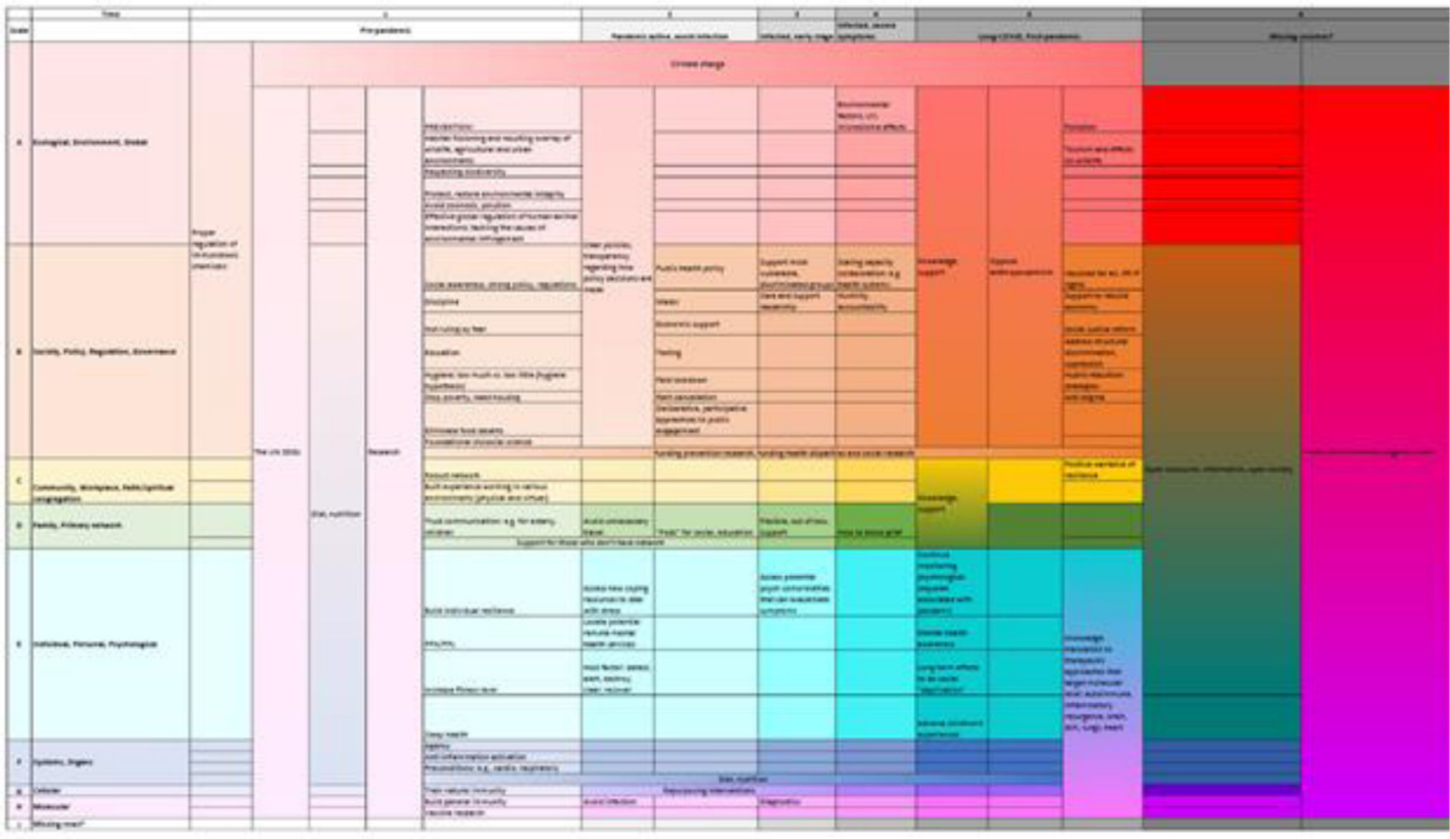
The Multiscale Health Action Matrix was a collaborative effort by the Multiscale Impact WG to begin to chart the full range of spatiotemporal scales and factors that need to be considered to understand and respond to COVID-19 and future pandemics The spatial scales (Y-Axis) range from the atomic/ molecular to systems, individuals, society and global-ecological. Temporally (X-Axis), the key events and factors precede the pandemic and personal infection. The timeline extends into the future to include long-COVID and post-pandemic considerations, thus reflecting the cyclical challenge of pandemic conditions.

The first meeting (“What”) consisted of surveying KEs and factors beyond the traditional molecular pathways. Meeting #2 (“How”) focused on the evolution of the AOP framework and new forms of visualization. Meeting #3 (“Why”) was a return to the basic tenet that what matters most is actual world impact and the forwarding of solutions including the assembly of a COVID/Pandemic Survival Kit. All meetings included a participatory *tour de table* and the use of collaborative tools such as polling, drawing, and chat. A main outcome culminated in Meeting #4, where multiple factors and their potential interactions across scales were consolidated in a Multiscale Health Action Matrix (Fig. 5).

#### Multiscale factors and outcomes.

Outcomes encompassed identification of KEs and factors that are not currently considered in AOPs, and widespread pandemic analysis. Examples included the cross-intersectionality of environmental scale effects, exposure to chemicals, individual and community resilience, diet and nutritional status, other animals, viral distribution, under-studied channels of transmission, life-styles, syndemics, psychosocial stress, government policy, and social justice. To this end, there was discussion regarding disparities including poverty, living/working density, health care, occupational exposure, knowledge and awareness, among many other factors.

#### Toward multiscale prevention.

Perhaps most importantly was the concern that we should be thinking beyond responses, and even resilience, toward prevention. One radical way to view this is that the SARS-CoV-2 virus should not be considered the initial KE. Rather, by looking across scales, multiple preceding causal, spatial and temporal factors could be identified, and their avoidance could have helped prevent the pandemic. In particular, there is a need to turn our attention to interactions with other species at an ecological and personal level. This includes human ecological damage, industrial food production, and laboratory practices that could all be nexuses for initial zoonosis events and pandemic-spread intensifiers.

#### New multiscale perspectives of AOPs.

An analysis of the dissatisfaction with the current AOP framework led to the identification of the need to develop concepts and tools to address the multi-scale aspects: explicit representation of time, simultaneity, multi-scale events, multi-system interactions, causality, nonlinearity, recurrence, and intensity of effects, as well as beneficial outcomes.

#### Future directions.

Next steps include evolving KEs, the Wiki, and new tools to better accommodate a dynamic multiscale perspective as well as (auto)ethnographic reflections on the process, community collaborations, novel creative approaches, informational handouts, and academic publications.

### 2.8 Integrated findings from 3 WGs

Gillina Bezemer then presented the integrated results of the toll-like receptor (TLR) endeavors across 3 WG (Hub/Lung-, MF-and Multiscale). She underlined that the outcome of exposure to SARS-CoV-2 and TLR stressors can be adverse, neutral or beneficial depending on various MFs of host and environment (Bezemer and Garssen, 2021). In analogy to Paracelsus’ basic principle of toxicology, “the dose makes the poison”, she summarized this multifactorial phenomenon as “the context makes the poison” or, more specifically, “the dose in the context differentiates a poison and a remedy”. By using the specific TLR example, she illustrated that a dual outcome can in part be captured within AOPs (AOP378, AOP377 - Table 4: work in progress in the AOP Wiki), and in part within beneficial outcome pathways (BOPs). The first BOP example (BOP1) shares KE and KER with AOP377, but in contrast describes a pathway of TLR9 activation, leading to a beneficial outcome in the specific context of allergic asthma. Combining insights from AOP and BOP could help to fill knowledge gaps, reveal novel treatment strategies, and shed light on potential side effects of treatments. A publication is planned to elaborate further on the idea of a BOP concept to facilitate organization of knowledge of health promoting substances and compounds, host factors, intervention initiating events (IIE), and prevention initiating events (PIE).

## 3 The CIAO debate

The COVID-19 pandemic being multifaceted, a CIAO debate was organized during the workshop to generate interactive discussions on “*Biology or society: which impacts COVID-19 most?*”. In a poll taken prior to the debate, 59% of workshop participants voted for biology, and 33% voted for society. Taking turns, Elan Ohayon, arguing for social factors, and Gillina Bezemer, supporting biology, gave their opinions and rebutted opposing ones from each other and from the audience. Social factors enumerated included disparities, occupational exposure, density, geo-political, health access and social justice issues, reflecting multiple forms of discrimination including racism and poverty. It was argued that these human social actions result in AOs ranging from psychosocial stress to ecological destruction and zoonosis. Conversely, identifying these social factors could lead to positive actions including physical distancing, wearing masks, testing capacity, government policy and open science. The “Taiwan-Index” case was used to illustrate the efficacy of social approaches. For biology, the relevance of biological knowledge of viruses and hosts was highlighted for three pillars: control, prevention and management of COVID-19. Examples included routes of transmission, diagnostic assays, biomarkers, genetic and lifestyle factors affecting immune responses and biological age, vaccines, and pharmaceutical treatments. At the end of the debate, another poll was taken, with the result being 45% voted for biology and 45% for society, reflecting that opinions changed but also the false dichotomy of the initial question, as both biology and society influence COVID-19 outcomes.

## 4 Interdisciplinary expertise: expanding the CIAO network

On the first day of the workshop, along with the scientific findings, logistical challenges faced by the WGs were presented, such as practical applications of the CIAO outputs still being unclear and needs for more resources as well as for specific expertise. The second day was dedicated mainly to addressing these challenges, as well as how to integrate issues related to virus variants and long-term aspects (long COVID) into the project. The best way to re-use AOP elements (KE and KERs) was also addressed. Clemens Wittwehr presented plans for an outreach campaign scheduled to start in May-June that will aim at attracting more contributing members to the crowd but also potential users of the CIAO knowledge. Following that, participants chose among several breakout (BO) groups to discuss the topics, then came back to present findings and discuss steps moving forward.

### 4.1 Breakout (BO) findings

#### BO 1: Integrate SARS-CoV2 variants and long COVID

This BO focused on understanding the effects and mechanisms of long COVID. There was some discussion about the potential evolution of the virus and the new variants appearing worldwide. That led to a suggestion to broaden the scope to vaccination issues, as well as the need for long-term longitudinal studies. The term “temporal phenotyping”, which refers to how a particular phenotype evolved over time, was suggested as an interesting way to explore COVID-19-related AOP networks. When considering the temporal aspect, factors such exposure, diet, etc. could be AOs or MFs. The spatial scale was also considered important and this could be particularly relevant for the different variants. The BO expressed interest in creating a 3D matrix and suggested the creation of a dynamic animation for AOPs to depict and understand temporal scales. The different scales would likely require the incorporation of different types of networks and approaches, both computationally and expert-driven. It would also be of great importance to consider how to organize and manage the data that is being gathered to develop AOPs.

#### BO 2: How to broaden the crowd

This BO group was tasked with discussing how to bring in more expertise to the CIAO project. It was discussed that expertise is needed in specific areas, particularly where evidence is contradictory. It may be suitable to determine expertise gaps systematically, e.g., through a survey disseminated to all CIAO participants. It may also be helpful to first determine what expertise we already have within each WG. A list of CIAO participants could be created along with brief biosketches (biographical sketches are used to describe an individual’s qualifications and experience). It was then discussed how novel expertise could be brought in in varying capacities. External experts could be brought in to advise the project on an *ad hoc* basis. Full-level participation still may be needed in some areas, which may require another round of CIAO crowd recruitment. Trainees with less experience could be offered well-defined work, allowing them to gain valuable research experience, authorship, and experience in participating in a global collaboration. The BO group then discussed how to identify expertise. Authors from relevant papers are an obvious choice. The CIAO crowd may also want to reach out to other groups focusing on COVID-19 (Annex B^5^), and the project should continue to be promoted at conferences. Lastly, there is a need to make a pitch selling the crowd’s broad expertise as well as the unique application of the AOP framework to COVID-19 pathogenesis and multiscale impact. It would help to have professional communications and promotional materials such as a website, social media and videos.

#### BO 3–4: Impact of CIAO and how to better reach the target audience

The BO group reflected on what added value the CIAO project would have for policy- and decision-makers. The group agreed that making the biology behind CIAO better understandable to the public would make individuals more receptive to COVID-related safety measures (wearing masks, quarantines, lockdowns, vaccinations) and social resistance to these measures would decrease, thereby supporting policy-makers.

The BO group reflecting on the added value of the CIAO outcomes for healthcare identified that COVID-19 AOPs could support clinicians to inform patients by describing in a simple but robust way the viral disease trajectory and the factors modulating it. AOPs can be personalized based on patient history, as age, sex, diet or co-morbidities have been identified as MFs influencing the outcome. Furthermore, COVID-19 AOPs positioned temporally along the disease course might be relevant to the identification of diagnostic markers of disease onset or progression, which correspond to discrete KEs.

#### BO 5: Re-use of AOP elements

**Fig. 6:**
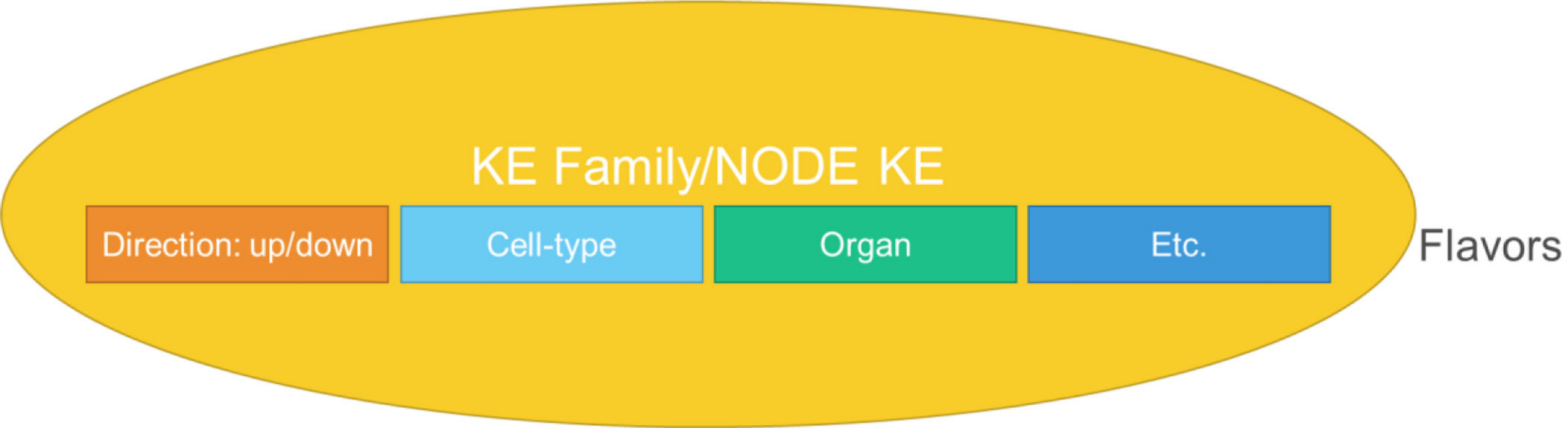
One possible future model of KE structure, where the KE would be grouped as a family or a “node”, e.g., ACE2 dysfunction, and different aspects of the KE would be encoded as sub-categories or “flavors” of the KE

The BO group discussing the challenges of re-using AOP elements focused on the need to maximize re-use to prevent proliferation of KE, to support the formation of interconnected AOP networks and to improve user interaction with the AOP Wiki. It was agreed that to achieve this it may be necessary to group similar or same KEs under one umbrella/family or as a “node” KE. In this vision, the KE is the general biological event (e.g., mitochondrial dysfunction, oxidative stress, inflammasome activation, ACE2 dysfunction) with sub-categories/layers/flavors being the direction of change and context (e.g., up-regulated, down-regulated, cell-type, organ, etc.) (Fig. 6). Within a particular context of use (e.g., addressing a research need, designing a testing strategy, building predictive models), the user can choose the level of specificity needed for the situation. The ability to include layers of specificity in such a structured way may help facilitate the organization and evaluation of the weight-of-evidence linking KEs, particularly the more complex ones. Such layering of specificity will also help with identification of appropriate assays and testing strategies to address AOPs. The group also discussed the importance of deciding what information belongs in a KE *versus* what goes into the KER. KERs are generally specific to a particular AOP and therefore already contain information specific to the AOP. So, it was recommended that as much of the generalizable information as possible goes into the KE. This is true in the current KE description approach as well as the proposed “layered” KE approach. A challenge to implementing this new “layered” KE structure will be to implement it at the coding level within the AOP Wiki. In addition, it will be a challenge to represent KER between these “layered” KEs, particularly at the coding level. In addition, it was recognized that optimal number and manner of representation of KEs has been particularly challenging due to unresponsiveness of some KE authors to participate in KE revisions. It was concluded that a discussion is needed on how to incentivize engagement of authors for the long term or find a solution for new developers when an issue arises, other than developing another discrete but similar AOP element.

### 4.2 Decisions made

The plenary then agreed that the WGs remain as they are (Tab. 5) and will continue to develop further AOPs, KEs and KERs. When relevant, information about SARS-CoV-2 variants and long-term aspects of COVID-19 will need to be integrated. The different WGs will establish a list of the expertise needed to consolidate their AOPs. The outreach campaign will be launched to attract those with the needed expertise and more CIAO crowd volunteers as well as scientific end-users. It was also agreed that a WG dedicated to writing the meta-level paper will also cover the application of the AOP framework to COVID-19 via crowdsourcing, as well as identifying case studies using contributions and achievements provided by the other WG to the CIAO Newsletter.

## 5 Conclusion and next steps

At the 3^rd^ CIAO AOP Design Workshop, the developed AOPs and KEs related to COVID-19 and entered into the AOP Wiki were presented and positioned within a timeline of the disease pathogenesis. New models of collaborations to broaden the crowd and case studies to reach the target audience were discussed in breakout groups. Challenges concerning the integration of MFs into the AOPs and AOP Wiki as well as issues related to the re-use of AOP elements (KE and KERs) were discussed.

Following the workshop, the WGs should focus on finalizing their AOPs/KEs/KERs/MFs and entering them into the Wiki while considering the impact of virus variants and long-term aspects (long COVID). The agreed publication strategy should be executed and the outreach campaign should be launched to broaden the scientific crowd, both COVID-19 AOP developers and users. Webinars for newcomers were planned for July 6 (1 pm CEST) and August. A workshop to initiate the meta-level paper was planned for June 30, and the first draft was expected by December 2021. The 4^th^ CIAO AOP Design Workshop was planned for September 2021 (Tab. 6).

Please visit https://www.ciao-covid.net/ if you would like to find out more, join the CIAO crowd or offer your expertise.

## Figures and Tables

**Tab. 1:** Hub and Lung KEs/AOPs

**Hub KEs**	KE1738	Susceptibility to viral entry, increased
	KE1739	ACE2 binding to viral S-protein
	KE1847	Coronavirus production, increased
	KE1854	ACE2 dysregulation
**Hub AOPs**	AOP379	Increased susceptibility to viral entry and coronavirus production leading to thrombosis and disseminated intravascular coagulation
	AOP392	Bradykinin and fibrinolytic dysregulation, hyperinflammation
**Lung AOPs**	AOP320	Binding of viral S-glycoprotein to ACE2 receptor leading to acute respiratory distress (ARDS) associated mortality
	AOP377	TLR9 activation leading to ARDS and Multi Organ Dysfunction
	AOP173	Substance interaction with the lung resident cell membrane components leading to lung fibrosis
	AOP319	Inhibition of Angiotensin-converting enzyme 2 leading to lung fibrosis
	AOP302	Lung surfactant function inhibition leading to decreased lung function

**Tab. 2:** Liver, heart and gut KEs and AOPs

**Liver** – *Indirect* AOPs		
Hub AOP	AOP379	Increased susceptibility to viral entry and coronavirus production leading to thrombosis and disseminated intravascular coagulation
Liver KE	KE1549	Liver injury
Liver AOP	TBD	Viral entry in lungs leading to thrombosis resulting in liver injury

Hub AOP	AOP392	Bradykinin and fibrinolytic dysregulation, hyperinflammation
Liver KE	KE1549	Liver injury
Liver AOP	TBD	Systemic inflammation resulting in liver injury

**Liver** – *Direct* AOP		
Hub KEs	KE1739	ACE2 binding to viral S-protein
	KE1738	Susceptibility to viral entry, increased
Liver KEs	KE902	Liver inflammation
	KE1549	Liver injury
Liver AOP	TBD	Binding of SARS-CoV-2 to ACE2 receptors expressed on cholangiocytes leads to liver inflammation resulting in liver injury
**Heart** - *Indirect* AOP		
Hub AOP	AOP392	Bradykinin and fibrinolytic dysregulation, hyperinflammation
Heart KEs	TBD	Myocardial injury
	KE1535	Heart failure
Heart AOP	TBD	Systemic inflammation resulting in heart failure

**Gut -** *Indirect* AOPs		
Hub AOP	AOP379	Increased susceptibility to viral entry and coronavirus production leading to thrombosis and disseminated intravascular coagulation
Gut KE	TBD-X	GI disorders
Gut AOP	TBD	Viral entry in lungs leading to thrombosis resulting in GI disorders

Hub AOP	AOP392	Bradykinin and fibrinolytic dysregulation, hyperinflammation
Gut KE	TBD-X	GI disorders
Gut AOP	TBD	Systemic inflammation resulting in GI disorders

**Gut -** *Direct* AOP		
Hub KEs	KE1739	ACE2 binding to viral S-protein
	KE1854	ACE2 dysregulation
Gut KEs	TBD-Y	Intestinal permeability, increased
	TBD-X	GI disorders
Gut AOP	TBD	Binding of SARS-CoV-2 to ACE2 receptors expressed on enterocytes leads to intestinal hyperpermeability resulting in GI disorders

**Tab. 3:** Neuro KEs and AOPs

Hub KE	KE1739	ACE2 binding to viral S-protein
Neuro KEs	KE188	Neuroinflammation
	KE352	Neurodegeneration
	KE1841	Encephalitis
Neuro AOP	AOP374	Binding of SARS-CoV-2 spike protein to ACE2 receptors expressed on brain cells leads to neuroinflammation resulting in encephalitis

Hub KEs	KE1739	ACE2 binding to viral S-protein
	KE1738	Susceptibility to viral entry, increased
Neuro KEs	KE1870	Sustentacular cells, decreased
	KE1871	Olfactory sensory neurons, decreased
	KE1872	Olfactory epithelium degeneration
	KE1873	Impaired olfactory function (anosmia)
Neuro AOP	AOP394	SARS-CoV-2 infection leading to impaired olfactory function (anosmia)

Hub KEs	KE1739	ACE2 binding to viral S-protein
	KE1738	Susceptibility to viral entry, increased
Neuro KEs	KE1874	Blood brain barrier disruption
	KE1875	Cerebrovascular disease (stroke)
Neuro AOP	AOP395	Binding of SARS-CoV-2 spike protein to ACE 2 receptors expressed on pericytes leads to intravascular coagulation resulting in stroke

**Tab. 4:** Dual outcome of TLR endeavors captured within AOPs and BOP

KE1848	TLR dysregulation
AOP378	Impaired TLR function leading to high pathogen load
AOP377	TLR9 activation leading to ARDS and Multi Organ Dysfunction
BOP1	TLR9 activation leading to less eosinophilic inflammation and improved lung function

**Tab. 5:** Current CIAO working groups (WG)

Working group name	Focus
Hub and Lung AOP group	KEs common to multiple COVID-19 AOs (e.g., coagulation, hyperinflammation) joint with pulmonary AOPs
Other Organs AOP group	AOPs and KEs specific to liver, kidney, heart, and gut
Neuro AOP group	AOPs and KEs linked to neurological impacts (anosmia, seizures, epilepsy, encephalitis, multiple sclerosis, blood-brain barrier...)
Modulating Factors group	Integrate modulating factors on KE/KER/AOP
Multiscale Impact group	Elucidate the multiscale factors of COVID-19 across levels and time and evolve the AOPs to address those multiscale aspects
Literature Review group	Applying systematic literature review to support AOP development
Meta-level paper group	Evaluate how the AOP framework and the crowdsourcing effort were applied to the disease area

**Tab. 6:** Next steps for CIAO

	Next steps	Timing
COVID-19 AOP network	Consolidation of AOPs/KEs within the WG	May-September 2021
	AOPs and KEs into the Wiki	May-September 2021
	KERs in the Wiki	May-September 2021
	Modulating factors in the KEs	May-September 2021
	AOP network publication - draft	December 2021
	Multi-scale approach	May-September 2021
	Literature review - protocol publication	May-September 2021
CIAO network	Outreach campaign	June 2021
	Webinars newcomers	July 6, 2021 – September 7, 2021
	Meta-level publication - workshop - draft	June 30, 2021 December 2021
	4th CIAO AOP Design Workshop	September 15–16, 2021

## Footnotes

1
https://aopwiki.org

2
https://www.ciao-covid.net

3
https://slack.com/

4
https://www.zotero.org/

5 doi:10.14573/altex.2112161s

6
https://www.oecd.org/officialdocuments/publicdisplaydocumentpdf/?cote=env/jm/mono(2016)12&doclanguage=en
